# Double-layered reconstruction of the nasal floor in complete cleft deformity of the primary palate using superfluous lip tissue

**DOI:** 10.1186/s40902-015-0035-z

**Published:** 2015-10-13

**Authors:** Young-Wook Park, Kwang-Jun Kwon, Min-Keun Kim

**Affiliations:** Department of Oral and Maxillofacial Surgery, College of Dentistry, Gangneung-Wonju National University, 7 Jukheon-gil, Gangneung, 210-702 South Korea

## Abstract

After cleft lip repair, many patients suffer from nasolabial fistulas, asymmetrical nasal floor, or an indistinct nostril sill, as well as intraoral wound dehiscence and subsequent scar contracture of surgical wounds leading to vestibular stenosis. For successful primary nasolabial repair of complete cleft deformity of the primary palate, cleft surgeons need special care in reconstructing the sound nasal floor. Especially when the cleft gap is wide or when any type of nasoalveolar molding therapy was not performed, three-dimensional reconstruction of the nasal floor is critical for a balanced nasal shape. In this study, the author describes an effective method for reconstructing a double-layered nasal floor using two mucosal flaps from both sides of the fissured upper lip. This is a report of six patients with unilateral or bilateral complete cleft of the primary palate with a detailed description of the surgical technique and a literature review.

## Background

Cleft lip and palate are the most common congenital orofacial anomalies that are treated by maxillofacial plastic and reconstructive surgeons. Successful surgical treatment requires delicate surgical skill, profound knowledge of abnormal anatomy, and a thorough understanding of three-dimensional orofacial esthetics. Well-performed cheiloplasty provides lifelong self-esteem to patients and their parents.

However, despite primary lip repair, a regrettable or unsatisfactory outcome often results when the patient becomes an adult. These secondary deformities after primary lip repair include lip deformities, nasal deformities, and oronasal fistulas [1, 2]. Among these, oronasal fistula and the severity of a cleft lip nasal deformity are closely related to the completion of a successful nasal floor reconstruction, especially when the cleft deformity is complete and wide. Clinically, it is important to minimize the secondary deformities after primary cheiloplasty. Repair of a secondary nasal deformity remains a special challenge that is best treated by preventive surgery at the time of primary cheiloplasty.

A poorly formed nasal floor not only results in an oronasal fistula and an unbalanced nasal shape but also fails to preserve mucosal tissues for coverage of the alveolar cleft that is performed at a later stage. However, some cleft surgeons often pay little attention to reconstruction of the nasal floor during primary lip repair. The aim of this study is to report a technique used by the author that can be referred to as “double-layered reconstruction of the nasal floor.” This technique results in a full nasal floor and nostril sill as well as intraoral mucosal coverage without leaving any defects in the wide cleft lip.

## Case presentation

**Fig. 1:**
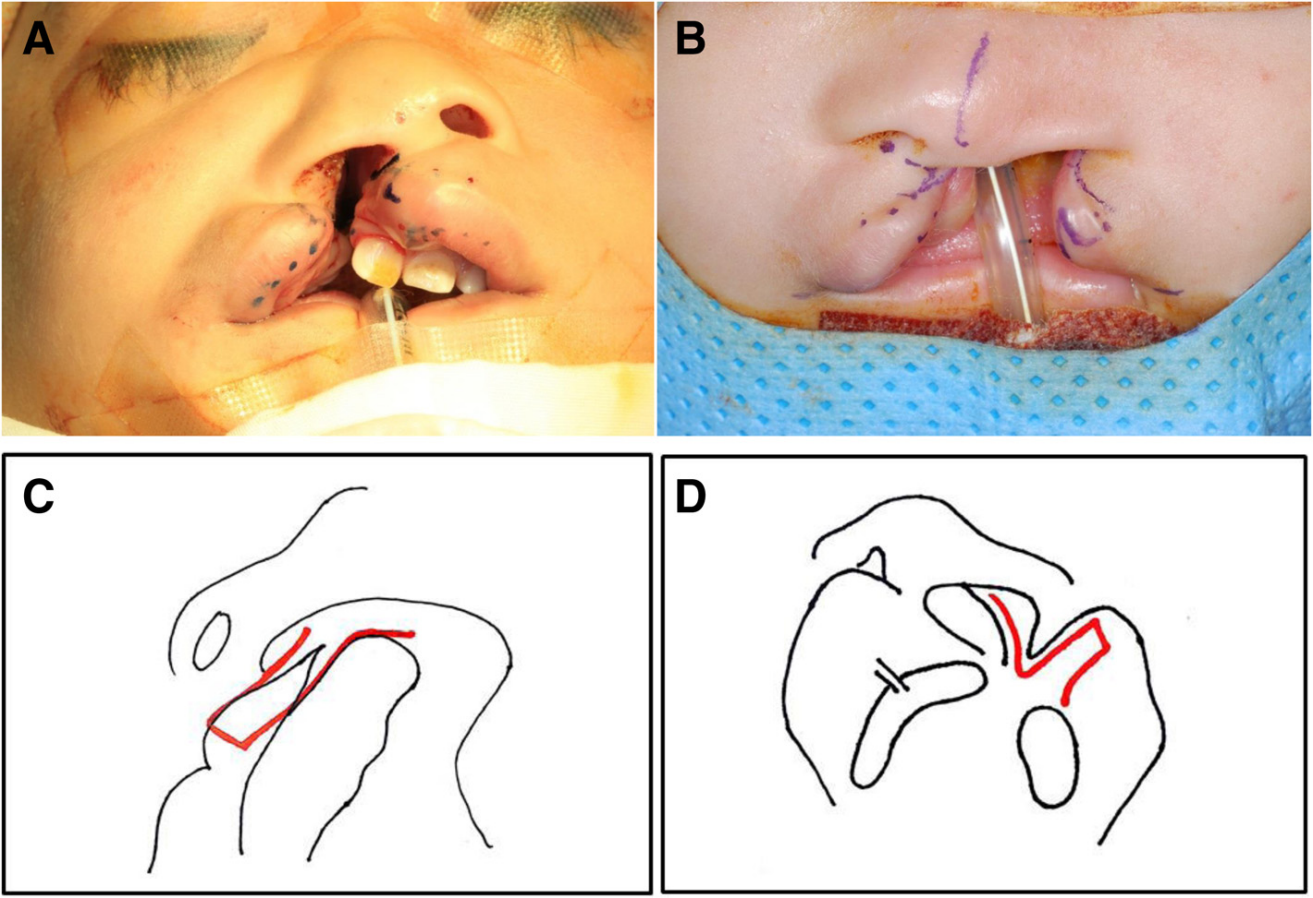
Preoperative marking and skin design for patients 1 (**a**) and 2 (**b**). Diagram of medial (**c**) and lateral (**d**) mucosal flaps for lining of the nasal floor

Six patients were included in this study. The patients’ basic information and diagnoses are listed in Table 1. All patients had no related malformations in other parts of the body. There had not been any previous treatment to mold the alveolar segments or the lower lateral cartilages of the cleft nose. Nasolabial repair was performed under general anesthesia with orotracheal intubation. The points and skin incisions basically followed Millard’s principle with addition of a small triangular flap in the lateral lip segment for unilateral complete cleft of the primary palate. Additionally, a dry vermilion flap was used when the thickness of the red lip vermilion was not matched between both sides of the upper lip. (Fig. 1a, b).

**Table 1:** Patients’ information and diagnosis

No.	Age (months)	Sex	Diagnosis
1	18	Male	Unilateral (right) complete cleft of primary and secondary palates
2	6	Female	Unilateral (left) complete cleft of primary and secondary palates
3	7	Female	Unilateral (right) complete cleft of primary palates
4	18	Female	Unilateral (right) complete cleft of primary and secondary palates
5	8	Male	Bilateral complete cleft of primary and secondary palates
6	14	Female	Bilateral complete cleft of primary and secondary palates

Basically, this technique involves the design of two mucosal flaps, one medial and the other lateral to the fissure (Fig. 1c, d). After the skin incision, two mucosal flaps were raised from the fissured red vermilion. For the preparation of the medial mucosal flap, the mucosal incision continued perpendicularly to the white roll and then toward the gingivobuccal sulcus. After completion of the incision, the submucosal dissection proceeded until the vestibular sulcus was reached. From there, the dissection was continued submucoperichondrially following the line where the septal mucosa and the base of the greater alveolar segment met until the vomer was reached. As a result, a rectangular-shaped mucosal flap which pedicled at the vomerine mucosa and a septal mucosal flap were elevated.

The lateral mucosal flap was raised from the lateral segment through the mucosal incision, which continued until the alveolar arch was reached. The skin incision followed the line where the lateral nostril base and nasal mucosa met. From this point, the incision was extended to the pyriform aperture. Via submucosal and subperiosteal dissection, a rectangular-shaped mucosal flap, which was pedicled at the pyriform aperture mucosa, and an alar base flap were elevated.

**Fig. 2:**
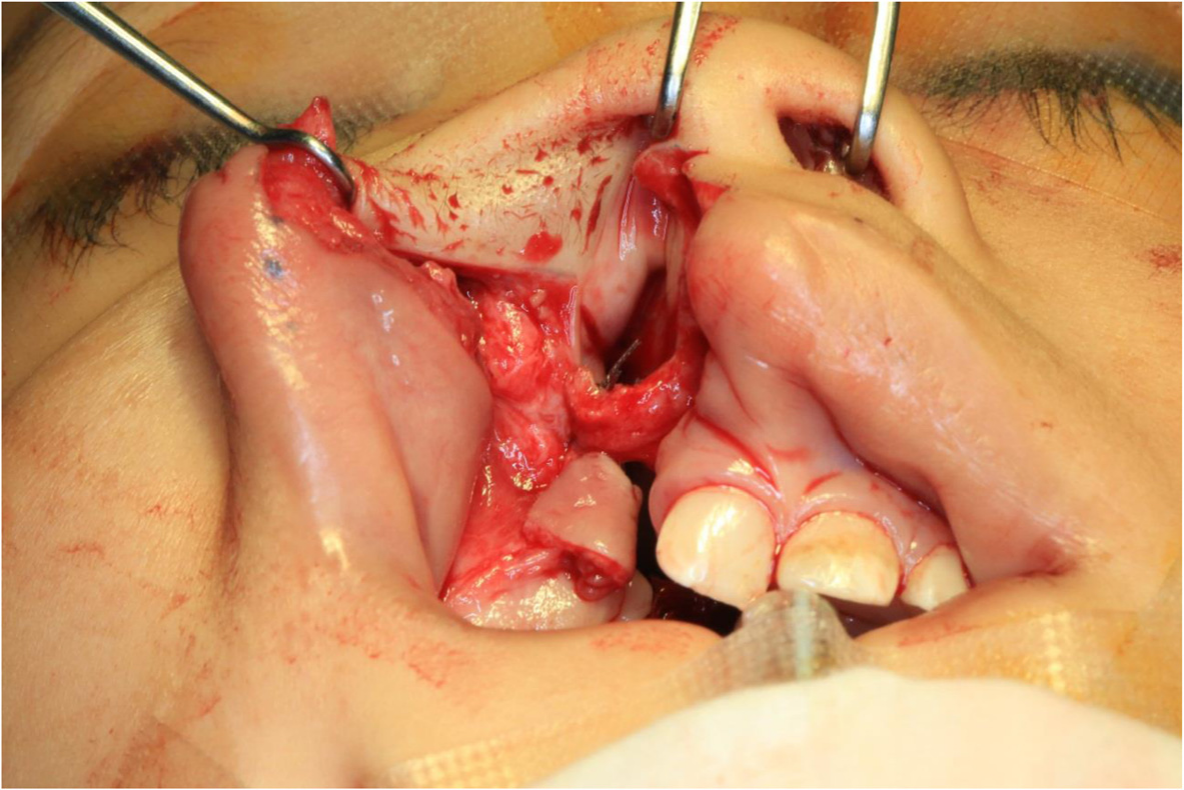
The medial mucosal flap, which is pedicled at the vomerine mucosa, was turned laterally by 90° and approximated to the lateral nasal mucosa with its mucosal surface to the nasal side

**Fig. 3:**
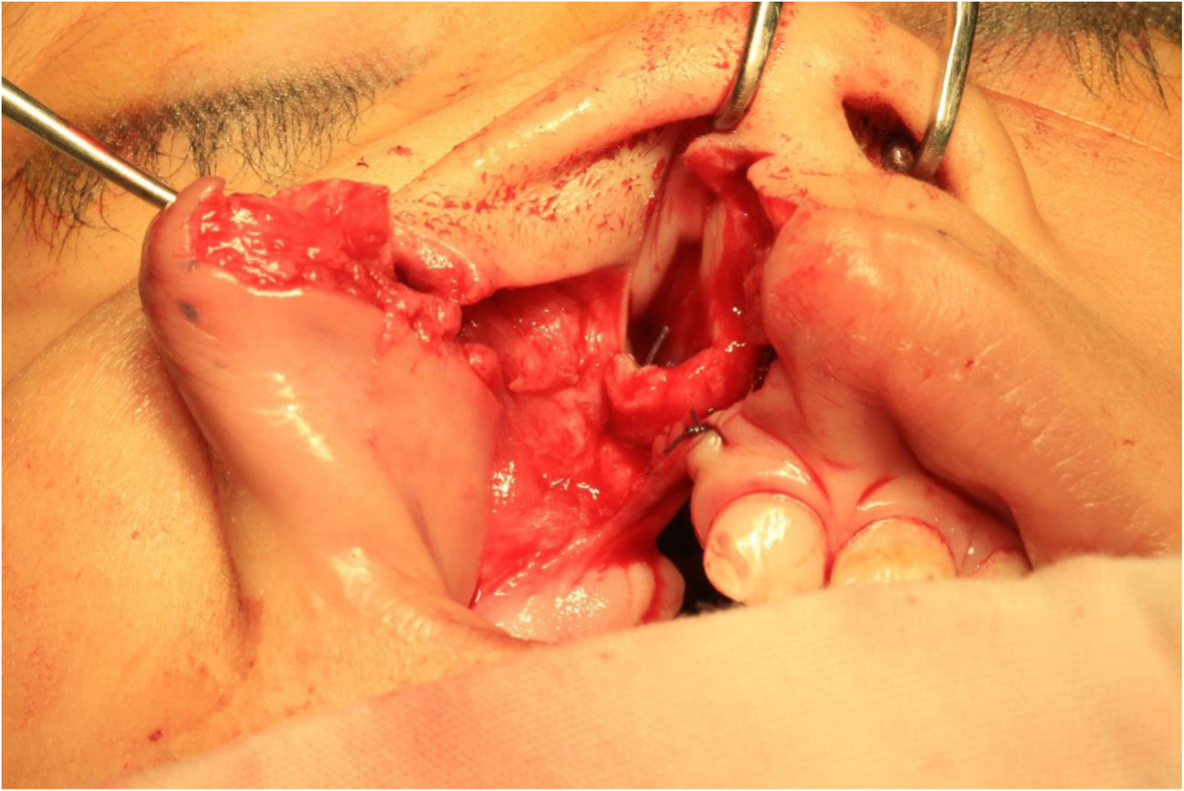
The lateral mucosal flap, which was pedicled at the root of the pyriform aperture, was turned medially by 90° with its mucosal surface to the oral side. It was approximated to the tissue cuff of the gingivopalatal mucosa on the edge of the alveolar cleft

**Fig. 4:**
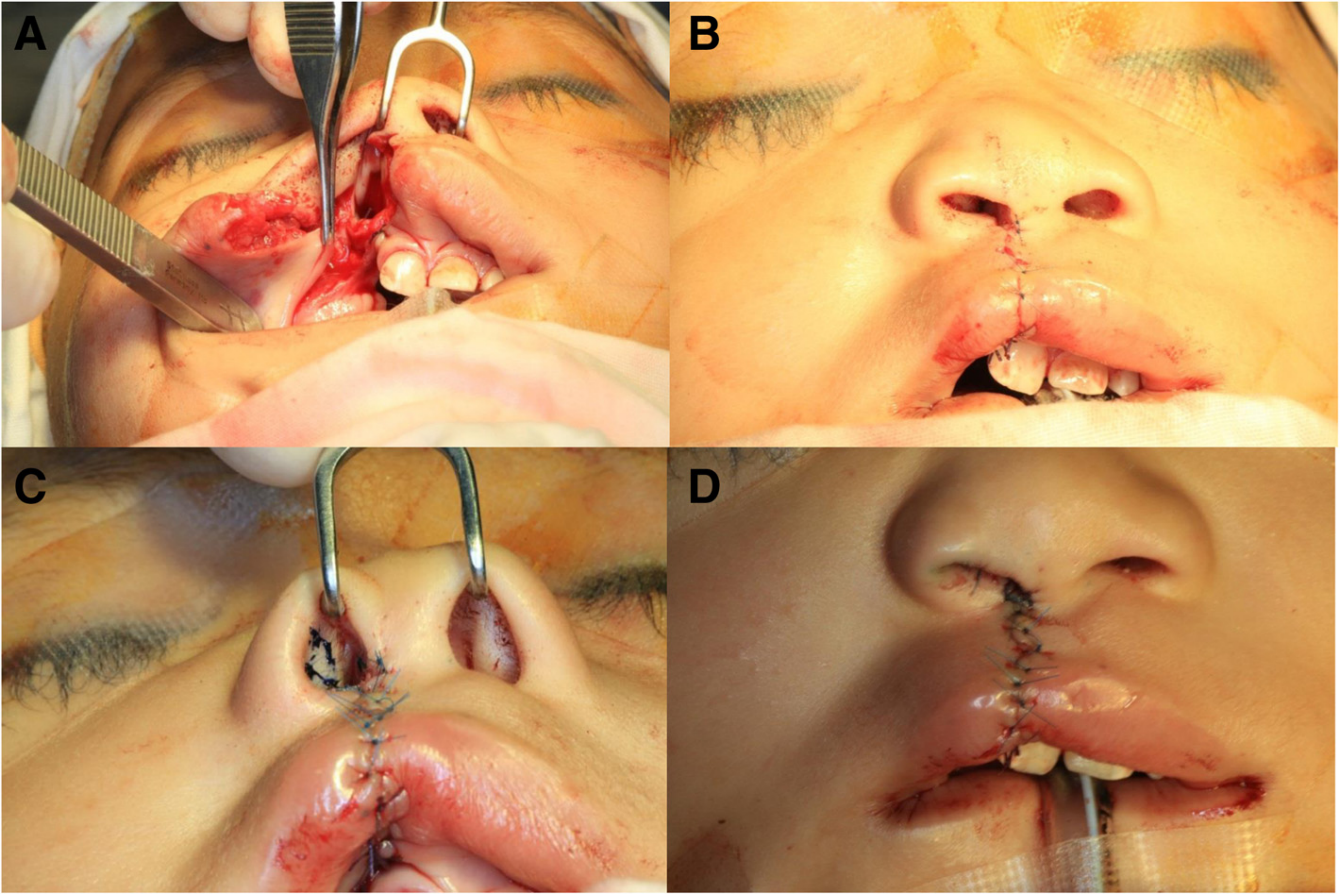
**a** Lateral vestibular mucosa was widely dissected and fixed to the anterior edge of the lateral mucosal flap and the labiogingival mucosa of the medial alveolar segment. **b** Alar base flap as approximated to the septal flap of the medial segment. **c** Nasal seal was made by fine sutures. **d** All skin sutures were performed

**Fig. 5:**
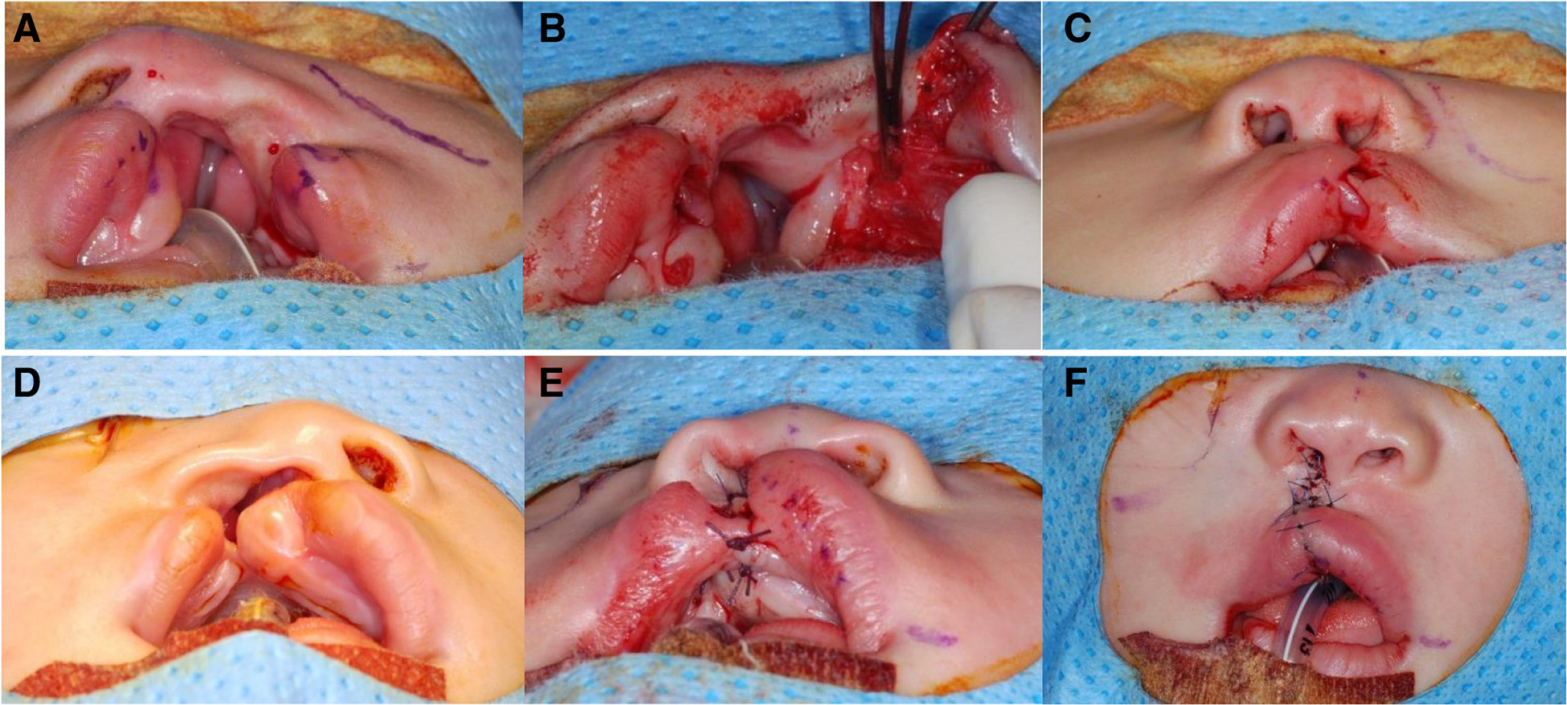
**a** Preoperative photogram of a 6-month-old female with a wide unilateral complete cleft of the primary and secondary palates (patient 2). **b** Intraoperative photogram of prepared mucosal flaps for reconstruction of the nasal floor. **c** Intraoperative photogram of symmetrical nostrils and full nasal sill. **d** Preoperative photogram of a 7-month-old female with unilateral complete cleft of the primary palate (patient 3). **e** Nasal floor was reconstructed by the prepared mucosal flaps. **f** Sutures at the end of the operation

The medial mucosal flap was horizontally rotated by 90°. It was sutured to the lateral nasal mucosa with its mucosal surface to the nasal side. This procedure formed the upper layer of the nasal floor (Fig. 2). After trimming some redundant tissues, the lateral mucosal flap was rotated horizontally by 90° with its mucosal surface to the oral side. It was sutured to the tissue cuff of the gingivopalatal mucosa on the greater alveolar segment to form the lower layer of the nasal floor (Fig. 3). The labial side of the fixed lateral flap was covered by the vestibular sliding flap, which forms the gingivolabial groove near the alveolar gap (Fig. 4a). After repositioning of the orbicularis oris muscle, the alar base flap was approximated to the septal flap, which forms the nostril floor and nasal sill (Fig. 4b–d). At this point, complete liberation and rotation of the lateral alar prevented deformation of the nostril. Similarly, the nasal floor was reconstructed in a double-layered fashion and the intranasal and intraoral raw surfaces were covered by sound tissues. Postoperatively, the symmetry of nostril shape and the fullness of the nostril sill were achieved (Fig. 5).

**Fig. 6:**
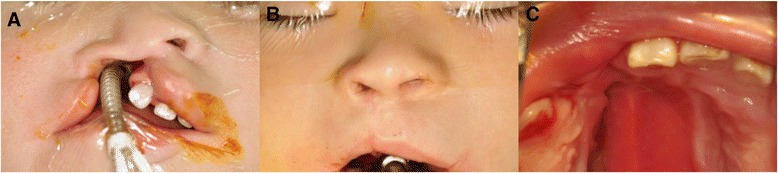
**a** Preoperative photogram of an 18-month-old female with wide unilateral complete cleft of primary and secondary palates (patient 4). **b** Postoperative photogram at 1 year after nasolabial repair with double-layered reconstruction of the nasal floor. **c** Mucosal tissue covers alveolar gap to the level of the incisive foramen without any nasolabial fistula or defect

Patient 4 showed a severe cleft lip and nasal deformity at initial diagnosis because she had not received hospital care until the nasolabial repair was performed. At 18 months after birth, the nasolabial repair with reconstruction of a double-layered nasal floor was performed. One year after the operation, her secondary nasal and nostril deformities were minimal. Intraorally, sound mucosal tissues covered the alveolar gap of the cleft alveolus completely to the level of the incisive foramen (Fig. 6).

**Fig. 7:**
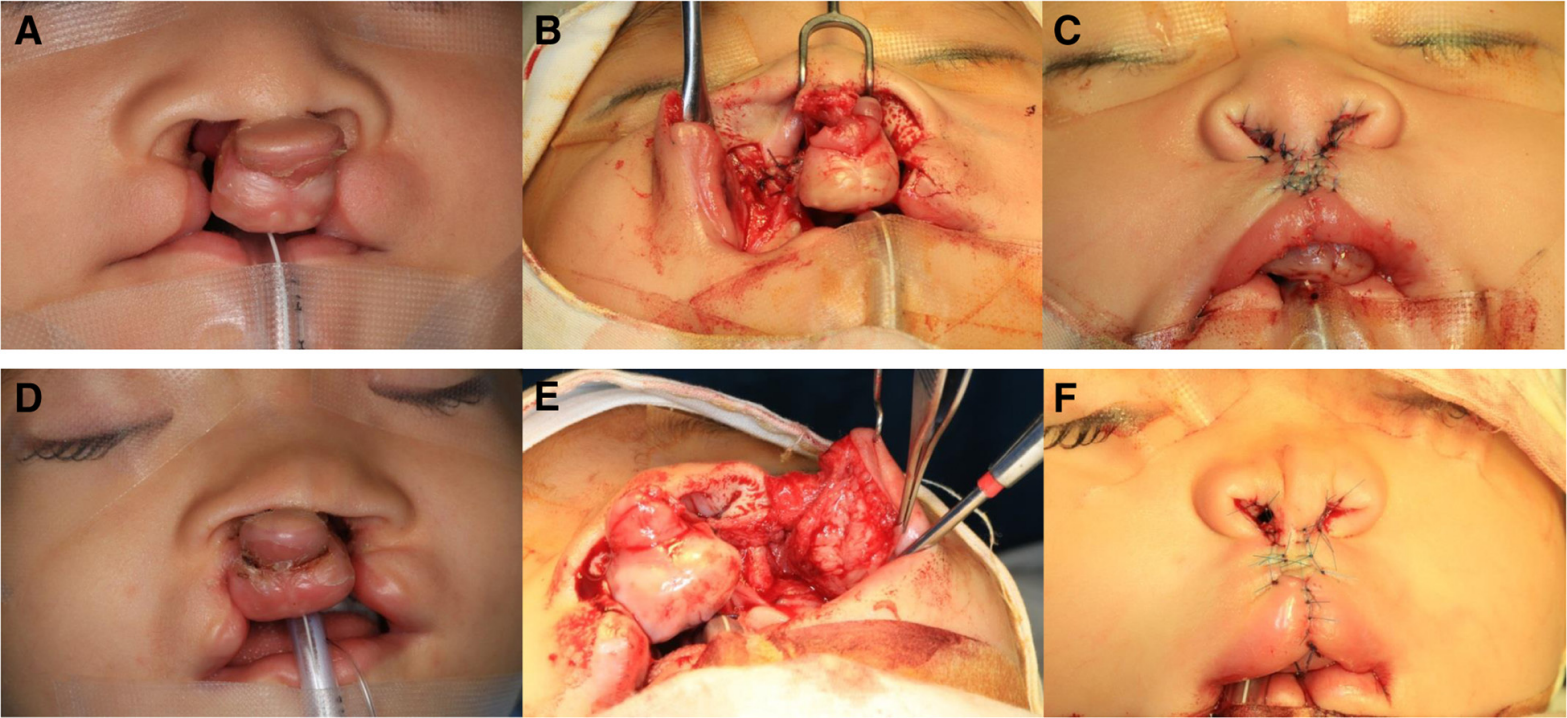
**a** Preoperative photogram of an 8-month-old male with bilateral complete cleft of primary and secondary palate (patient 5). **b** Intraoperative photogram of double-layered reconstruction of the nasal floor. **c** All skin sutures were performed. **d** Preoperative photogram of a 14-month-old female with bilateral complete cleft of primary and secondary palates and severely displaced premaxilla (patient 6). **e** After repositioning of the displaced premaxilla by vomer ostectomy, the nasal floor was reconstructed by mucosal flaps. **f** All skin sutures were performed

In repairing the bilateral complete cleft lip, the same concept was applied to the reconstruction of the nasal floor. The markings and skin flap design followed Millard’s bilateral lip repair and added a rim incision on the superior nostril to perform a primary rhinoplasty. In the case of patient 6, the severely displaced premaxilla was repositioned via vomer ostectomy before nasolabial repair. After incisions and dissection, suturing began with the repair of the nasal floor. Lip repair was carried out in the conventional manner after radical mobilization and approximation of the orbicularis oris muscle (Fig. 7).

### Discussion

Recent use of preoperative orthopedics, such as nasoalveolar molding or more active Latham appliances, has helped cleft surgeons to perform a less aggressive gingivoperiosteoplasty in primary cheiloplasty. Recently, the incidence of nasolabial fistula has been reported in less than 1 % of surgeries after nasolabial molding therapy [3]. But, even now, many patients with cleft lip deformity still show a wide alveolar gap after they have had primary cheiloplasty.

In cleft lip repair, as the cleft gap is wider, esthetic reconstruction of nasolabial anatomic components becomes more difficult. If it is not wide, the undersurface of the repaired nostril floor usually is healed by the granulation tissue and is covered by mucosa on the oral side even if there are raw surfaces or surgical defects. The so-called wide cleft lip refers to when the ratio of the gap width to the upper lip length on the normal side is 8:10 or higher [4]. The patients in this study, except for number 3, showed a wide cleft lip. These patients were suitable candidates for the present technique, i.e., the double-layered reconstruction of the nasal floor.

The tissues used in this technique are considered to be natural superfluous lip tissues. From a certain point of view, the mucosal flaps in this study are very similar to the M and L flaps of Millard. Millard had proposed the M flap to cover the defect created by the intraoral mucosal back-cut incision for inner downward rotation [5]. This maneuver effectively elongated the medial lip segment. Currently, many cleft surgeons actually cut off this superfluous tissue because lip length can be maintained if muscular reorientation and draping are definitely performed. Actually, the medial mucosal flap in this technique is quite different from Millard’s M flap in its dimension and the usage. The extended intranasal incision that is continuous with the skin incision is the same, but the lower mucosal incision is extended intraorally along the gingivomedial sulcus of the greater alveolar segment to the incisive foramen. This flap is composed of basal nasal mucosa and caudal lip mucosa and used for the nasal floor posterior to the nostril floor. As a result, anatomically matched tissues are engaged in the reconstruction of the nasal floor.

Originally, Millard’s L flap was used to fill the pyriform aperture defect as the cleft alar base advanced into the ideal position [6]. However, this had a pitfall in that the blood supply for the L flap comes from the alveolus, thus cutting the modified Millard’s L flap so that the blood supply would come from the lateral nasal wall. He turned this flap upward by 180° and closed the pyriform aperture defect [7]. Chang et al. also used this type of lip mucosal flap in a wide unilateral complete cleft lip [4]. In the present technique, the base of the lateral mucosal flap is wider and the circulation comes from the alveolus and the nasal wall. Finally, the base of the lateral mucosal flap crosses the alveolar gap and reconstructs the nasal floor in a double-layered fashion.

The advantage of this technique is the prevention of a nasolabial fistula or an anterior palatal fistula in the wide cleft lip. Therefore, this technique reduces the number of secondary surgeries, which result in scar contracture and growth inhibition. In the past, use of the vomer flap reduced the incidence of nasolabial or nasovestibular fistulas from 74 to 29 % [8]. Additionally, the use of a vomer flap to cover the raw area on the nasal surface in cleft palate pushback was reported [9]. However, in recent concepts for cleft management, the reconstruction of the nasal floor is considered part of the primary nasolabial repair because it affects the nasal shape. Cutting et al. reconstructed the nasal floor of unilateral complete lip deformities in a double-layered fashion using the traditional mucosal turn-over flap [10], but such a flap is not always possible in wide cleft lips.

In bilateral cleft lip deformities, such as those in patients 5 and 6, the same technique was successfully applied. However, in the left side of number 6, which showed a very wide alveolar gap, the dimension of the medial mucosal flap was deficient in covering the alveolar gap and the surgical defect. That is because the vermilion of the prolabium usually shows atrophy. In these cases, the use of Noordhoff’s inferior turbinate flap [11] might be useful.

The second advantage of this technique is that it can significantly improve the esthetics of the cleft nose. Recently, primary cleft rhinoplasty has been accepted as a standard procedure for complete cleft lip deformity. Now, most cleft surgeons perform some form of primary rhinoplasty [12–14] because many clinicians have demonstrated that less traumatic surgical procedures are needed following primary cleft rhinoplasty. The goals of primary cleft rhinoplasty are reconstruction of the nasal sill and floor, repositioning of the alar bases, and creation of an increased nasal tip. Furthermore, in unilateral cleft lip rhinoplasty, formation of the symmetrical nostril contour is important. In this technique, the surgical dissection allows for a more anteriorly and superiorly positioned alar base and provides the internal mucosal sling which later supports the nasal shape. In this series of patients, although they had not undergone any type of nasoalveolar molding therapy, all showed full nasal floors and sills without any raw surfaces intraorally. Additionally, the author’s technique provides an additional dimension of the nasal floor posteriorly to the nostril floor. As a result, the size of the nostril base does not get smaller as it would be observed with the Millard repair.

The third advantage of this technique is that it uses the superfluous lip mucosal tissues, consequently providing sufficient mucosal tissues intraorally as shown in patient 4. In cleft lip repair, modern surgical techniques have been developed for anatomical realignment of the deformed tissues, which provide a foundation for future growth. We can use this superfluous mucosa in tension-free closure of surgical wounds for alveolar bone grafts, which are performed at a later stage. Therefore, double-layered reconstruction of the nasal floor is critical for better nasolabial esthetics, as well as for preservation of mucosal tissues in alveolar bone grafting.

## Conclusions

In summary, the author presents a surgical technique for the double-layered closure of the nasal floor in patients with unilateral and bilateral complete clefts of the primary palate. This is an effective surgical technique to reconstruct the nasal floor with anatomically suitable mucosal tissue. Furthermore, this technique yields more esthetic results in complete unilateral cases, and it is reproducible in patients with complete bilateral cases. In conclusion, the proposed technique might lead to a decreased incidence of nasolabial fistulas and asymmetrical nasal bases. It needs further long-term studies with a larger patient sample.

## Consent

Written informed consent was obtained from the patient for the publication of this report and any accompanying images.
